# Early experience with an opt-in research register - Scottish Health Research Register (SHARE): a multi-method evaluation of participant recruitment performance

**DOI:** 10.1186/s12874-021-01479-4

**Published:** 2021-12-20

**Authors:** Wen Shi, Shobna Vasishta, Louise Dow, Daniella Cavellini, Colin Palmer, Brian McKinstry, Frank Sullivan

**Affiliations:** 1 Population and Behavioural Science Division, School of Medicine, University of St Andrews, North Haugh, Fife, St Andrews, KY16 9TF UK; 2 Division of Population Health and Genomics, School of Medicine, University of Dundee, Dundee, UK; 3 Usher Institute, University of Edinburgh, Edinburgh, UK

**Keywords:** Electronic health records, Patient recruitment

## Abstract

**Background:**

Recruiting participants to a clinical study is a resource-intensive process with a high failure rate. The Scottish Health Research Register (SHARE) provides recruitment support service which helps researchers recruit participants by searching patients’ Electronic Health Records (EHRs). The current study aims to evaluate the performance of SHARE in participant recruitment.

**Methods:**

Recruitment projects eligible for evaluation were those that were conducted for clinical trials or observational studies and finished before 2020. For analysis of recruitment data, projects with incomplete data were excluded. For each project we calculated, from SHARE records, 1) the fraction of the participants recruited through SHARE as a percentage of the number requested by researchers (percentage fulfilled), 2) the percentage of the potential candidates provided by SHARE to researchers that were actually recruited (percentage provided and recruited), 3) the percentage of the participants recruited through SHARE of all the potentially eligible candidates identified by searching registrants’ EHRs (percentage identified and recruited). Research teams of the eligible projects were invited to participate in an anonymised online survey. Two metrics were derived from research teams’ responses, including a) the fraction of the recruited over the study target number of participants (percentage fulfilled), and b) the percentage of the participants recruited through SHARE among the candidates received from SHARE (percentage provided and recruited).

**Results:**

Forty-four projects were eligible for inclusion. Recruitment data for 24 projects were available (20 excluded because of missingness or incompleteness). Survey invites were sent to all the eligible research teams and received 12 responses. Analysis of recruitment data shows the overall percentage fulfilled was 34.2% (interquartile 13.3–45.1%), the percentage provided and recruited 29.3% (interquartile 20.6–52.4%) and percentage identified and recruited 4.9% (interquartile 2.6–10.2%). Based on the data reported by researchers, percentage fulfilled was 31.7% (interquartile 5.8–59.6%) and percentage provided and recruited was 20.2% (interquartile 8.2–31.0%).

**Conclusions:**

SHARE may be a valuable resource for recruiting participants for some clinical studies. Potential improvements are to expand the registrant base and to incorporate more data generated during patients’ different health care encounters into the candidate-searching step.

**Supplementary Information:**

The online version contains supplementary material available at 10.1186/s12874-021-01479-4.

## Background

It is a paradox that although most people are willing to participate in medical research, many research studies struggle to recruit participants [1]. Poor recruitment often lengthens studies, increasing the costs of research, and, at worst, results in underpowered inconclusive or abandoned studies [2]. One study in 2013 revealed that in the UK only 55% of studies achieved their target sample size and 45% required funding for a time-extension [3]. More recently, Tudur Smith et al. conducted an on-line Delphi survey of 48 clinical trials units (CTUs) and identified ‘*Research into methods to boost recruitment in trials*’ as the top priority in trial methodological research [4]. Although the challenge of participant recruitment has been well-recognized and frequently-stressed, strategies to deal with this problem currently are insufficiently evidence based. According to Treweek el al., only two approaches showed slight to moderate recruitment improvement with high-certainty evidence [5]. One of them is about adopting open rather than blinded placebo design and the other is to use a telephone reminder in addition to postal contact. Some researchers have turned to disease-specific registers to facilitate trial recruitment such as cancer register, diabetes register, which has obtained good results [6–9]. A more general approach – research register initiatives based on Electronic Health Records (EHRs) have been developed in different nations and countries with patient consent, e.g., ResearchMatch, [10] All of Us Research Program, [11] Discover-Now [12] and SHARE [13] and more recently without consent [14]. They provide recruitment support service by helping researchers identify eligible participants in the EHRs and recruit them into their studies.

SHARE, part of National Health Service Research Scotland, is an example of the EHR-based recruitment support service which seeks consent from people across Scotland for their EHRs to be utilized for identifying eligible participants for health care research [13, 15, 16]. The database was designed following consultation with patients and members of the general public [17]. Registrants are predominantly approached by SHARE in hospital waiting areas. As a result, the proportion of patients with long-term health conditions among SHARE members is higher than the general population. And that is corroborated by the data from the Scottish Burden of Disease Study 2016 [18] and the 2016 mid-year population estimate [19].

**Fig. 1:**
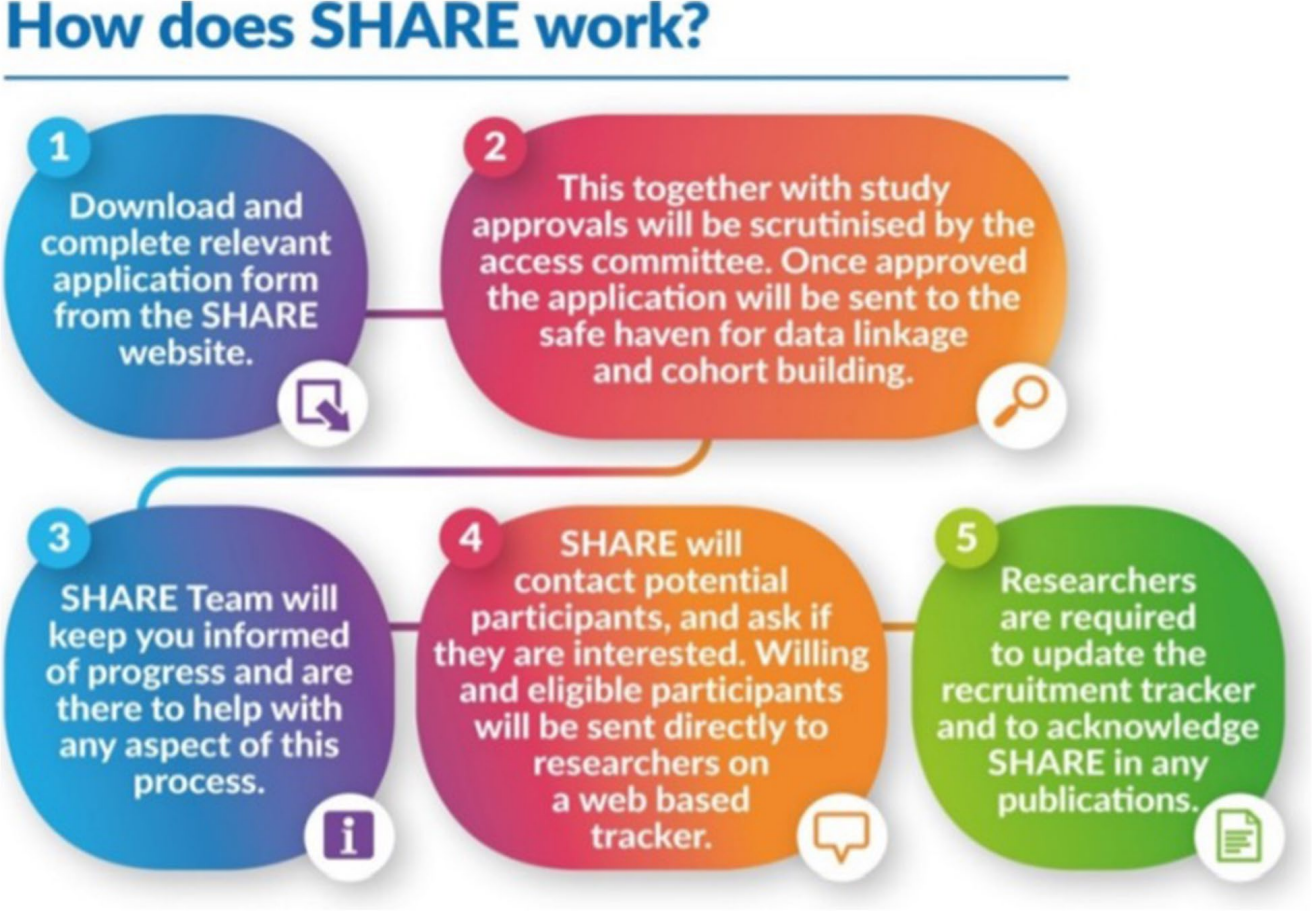
Workflow of recruiting through SHARE [13].

When a research team approaches SHARE for assistance with participant recruitment, the SHARE Studies Access Committee establishes that the study has ethical approval and that the number of participants requested is likely to be achievable. The Health Informatics Centre (HIC) then works with the researchers to create an optimal search algorithm to be implemented in the EHR database. HIC is a National Health Service Safe Haven where EHRs are hosted on a local server within a restricted, safe IT environment. Safe havens provide a platform for the use of NHS electronic data in research feasibility, delivery and pharmacovigilance [20, 21]. Currently the data most used are demographics, hospital admissions (SMR01), community drug prescriptions, laboratory test results and cancer register. The cohort derived from the database search are transferred to the SHARE tracker system. SHARE team contact those potentially eligible patients by phone, email or letter, provide information about the study and confirm their interest in participation. The SHARE tracker system contains contact information and a record of previous contacts and responses. SHARE registrants can set a contact cap which sets the maximum number of studies the registrants would like to be contacted about annually when they register with SHARE. Following contact, if potential candidates express an interest in the project then, with permission, their details are passed on to researchers for further eligibility screening and enrolment. Figure 1 shows the workflow of engaging with SHARE for participant recruitment.

Discover-Now, [12] ResearchMatch [10, 22] and ALL of Us [11, 23] are all similar recruitment support services. SHARE and Discover-Now are based in Scotland and England respectively while ResearchMatch and ALL of Us are national registers in the USA. Research Match is reported to have supported more than 700 studies [10, 22]. But so far there is no study which has explored the performance of these EHR-based participant recruitment methods in a systematic way.

The large SHARE registrant base has made it attractive to researchers. However, although SHARE has roughly been used for recruitment to 100 health research studies by 2020, it is still uncertain how successful a research infrastructure like SHARE is in terms of meeting recruitment targets and improving recruitment efficiency by reducing the number of people that researchers need to screen for different kinds of studies. Such information will help researchers decide if an EHR-based recruitment approach is likely to be successful for their type of study. It can also inform SHARE and the other research support groups of the strengths and weaknesses of the approach.

The current paper evaluates SHARE’s performance in participant recruitment for clinical studies and discusses how SHARE can improve in the future.

## Methods

### Registrant composition

The statistics of registrants’ characteristics were quoted from SHARE’s regular reports on age, gender, disease status and medication. Age and gender were recorded in the demographic dataset. Disease status was estimated based on the hospital admissions data and medication based on the community prescription data. The distribution of the characteristics was described with both raw numbers and percentages.

### Recruitment performance-data from SHARE

The SHARE team provided recruitment data for the included projects from their tracking records. To be included, studies must have completed the recruitment process in SHARE before 2020 and be either a clinical trial or an observational study. Projects were excluded from recruitment data analysis because SHARE could not provide recruitment data due to missing/incomplete records. Missing records were caused by projects cancelled by researchers due to personal or study specific reasons. Incomplete records happened to some projects which did not progress if the researchers were not satisfied with the initial search result. Recruitment data were also incomplete for projects in which SHARE was only requested to send out email advertisements and was not further involved in recruitment. For each included project, the total number of participants requested by researchers, the number of potentially eligible participants identified through searching EHRs, the number of potentially eligible participants provided by SHARE to researchers and the number finally recruited through SHARE were presented. Recruitment performance was indicated by three metrics, i.e., the fraction of the number of the patients recruited through SHARE over the total number requested (percentage fulfilled), the percentage of the recruited among the provided (percentage provided and recruited), the percentage of the recruited among the identified (percentage identified and recruited).

### Recruitment performance-survey of researchers

Since SHARE is often not the only source of recruitment, the data only reflect the recruitment requests and outcomes recorded by SHARE. It was considered worthwhile to conduct a survey of the SHARE users to investigate their study recruitment outcomes and their experiences of recruiting through SHARE. Thus, an online survey was conducted on Qualtrics [24] during Mar, 2020 and Apr, 2020 of the researchers of all the eligible projects which finished before 2020 for trials and observational studies. An email with a link to the online survey was sent to each researcher separately along with a participant information sheet. The researchers were asked how SHARE was involved in their studies, how they would rate SHARE for recruitment and their suggestions for improvement. They were also asked to report the target participant number of their study, the number of participants they received from SHARE, the number recruited through SHARE and through other recruitment methods as well. To ensure that researchers would be less likely to reject the survey due to worry about anonymity, all the survey questions were made optional, and the respondents could choose not to answer any question that they think might make them identifiable. The questionnaire and the participant information sheet are attached as Additional file 1. For each study which we received a response, we presented the raw numbers the researchers reported and calculated the fraction of the number recruited through SHARE over the total recruitment target (percentage fulfilled) and the percentage of the number recruited among the number received from SHARE (percentage provided and recruited).

### Analysis

Centre and variation of recruitment performance across studies were shown by median and interquartile range. All the calculations were done in the statistical software R(3.6.1) [25].

## Results

**Fig. 2:**
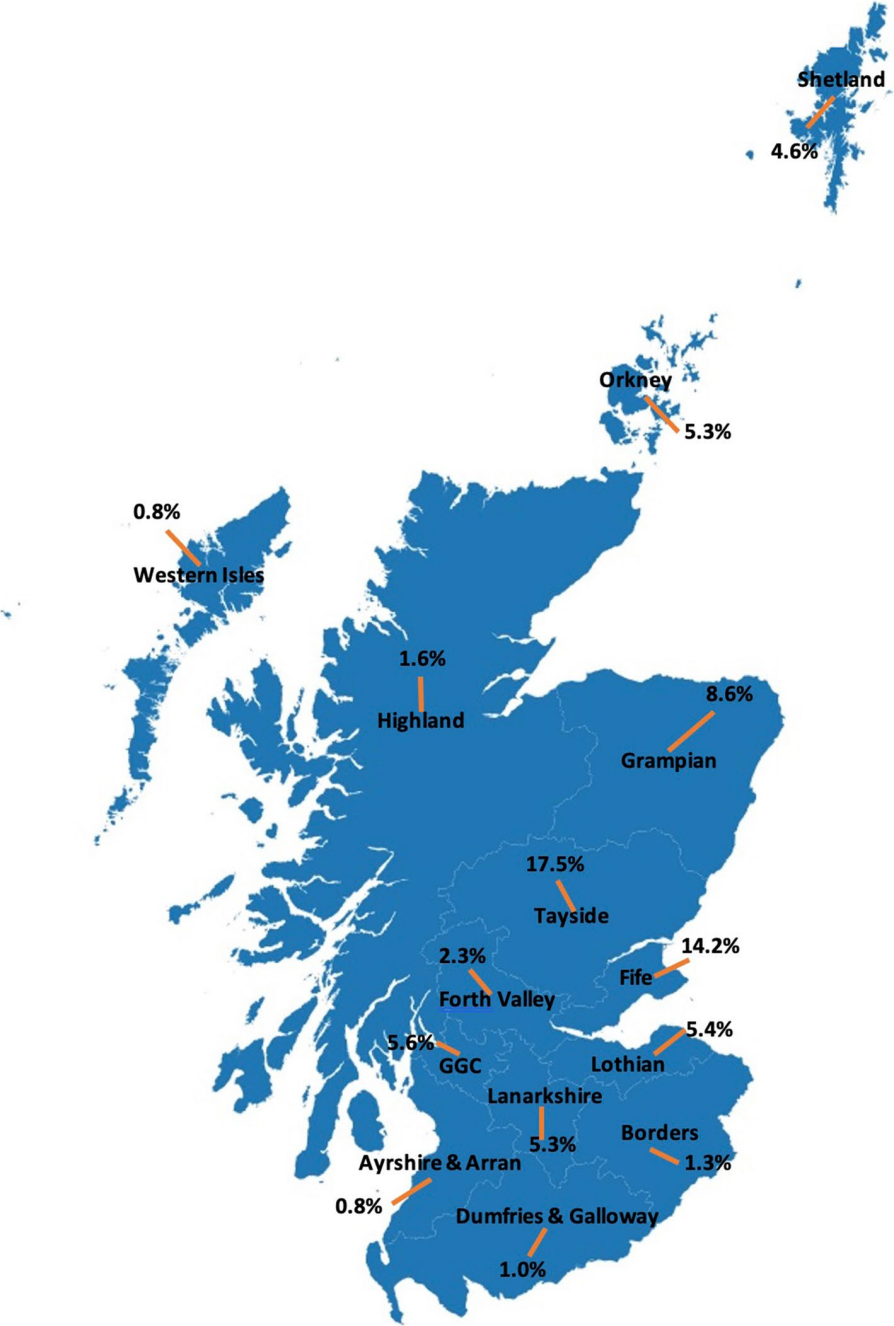
The proportion of SHARE registrants in the 16+ population in each health board across Scotland

As of August 2020, there were 283,791 patients registering with SHARE. The distributions of their age, gender, disease status and medication type are shown in the Table 1. Overall, 6.4% of the population over 16 in Scotland registered with SHARE. Figure 2 indicates the percentages of SHARE registrants in the 16+ population in each health board across Scotland.

**Table 1:** Distribution of characteristics of the SHARE registrants

Characteristics	Number of Registrants	Percentage
Age		
11–15	865	0.3
16–19	1654	0.6
20–29	20,130	7.1
30–39	30,492	10.7
40–49	34,678	12.2
50–59	52,509	18.5
60–69	58,775	20.7
70–79	55,791	19.7
80–89	25,457	9.0
90+	3440	1.2
Sex		
Female	170,459	60.1
Male	113,332	39.9
Disease status		
Any malignancy	20,124	7.1
Cerebrovascular disease	8734	3.1
Chronic pulmonary disease	22,454	7.9
Congestive heart failure	5329	1.9
Dementia	881	0.3
Diabetes with chronic complication	1571	0.6
Diabetes without chronic complication	18,476	6.5
Hemiplegia or paraplegia	1481	0.5
Metastatic solid tumour	4329	1.5
Mild liver disease	4575	1.6
Moderate or severe liver disease	1255	0.4
Myocardial infarction	9252	3.3
Peptic ulcer disease	4152	1.5
Peripheral vascular disease	5495	1.9
Renal disease	7449	2.6
Rheumatic disease	4862	1.7
Medication type		0.0
Analgesics	86,839	30.6
Antidiabetic Drugs	27,042	9.5
Antidepressant	72,201	25.4
Antihypertensives	89,418	31.5
Cardiovascular System	144,363	50.9
Central Nervous System	130,106	45.8
Endocrine System	80,870	28.5
Gastro-Intestinal System	170,990	60.3
Infections	92,156	32.5
Inhaled Steroids	29,588	10.4
Lipid Modifying Drugs	67,433	23.8
Muscoskeletal And Joint Disease	67,483	23.8
NSAIDs including Cox-2	35,408	12.5
Respiratory System	65,127	22.9
Skin	71,544	25.2
Topical Steroids	43,186	15.2

**Fig. 3:**
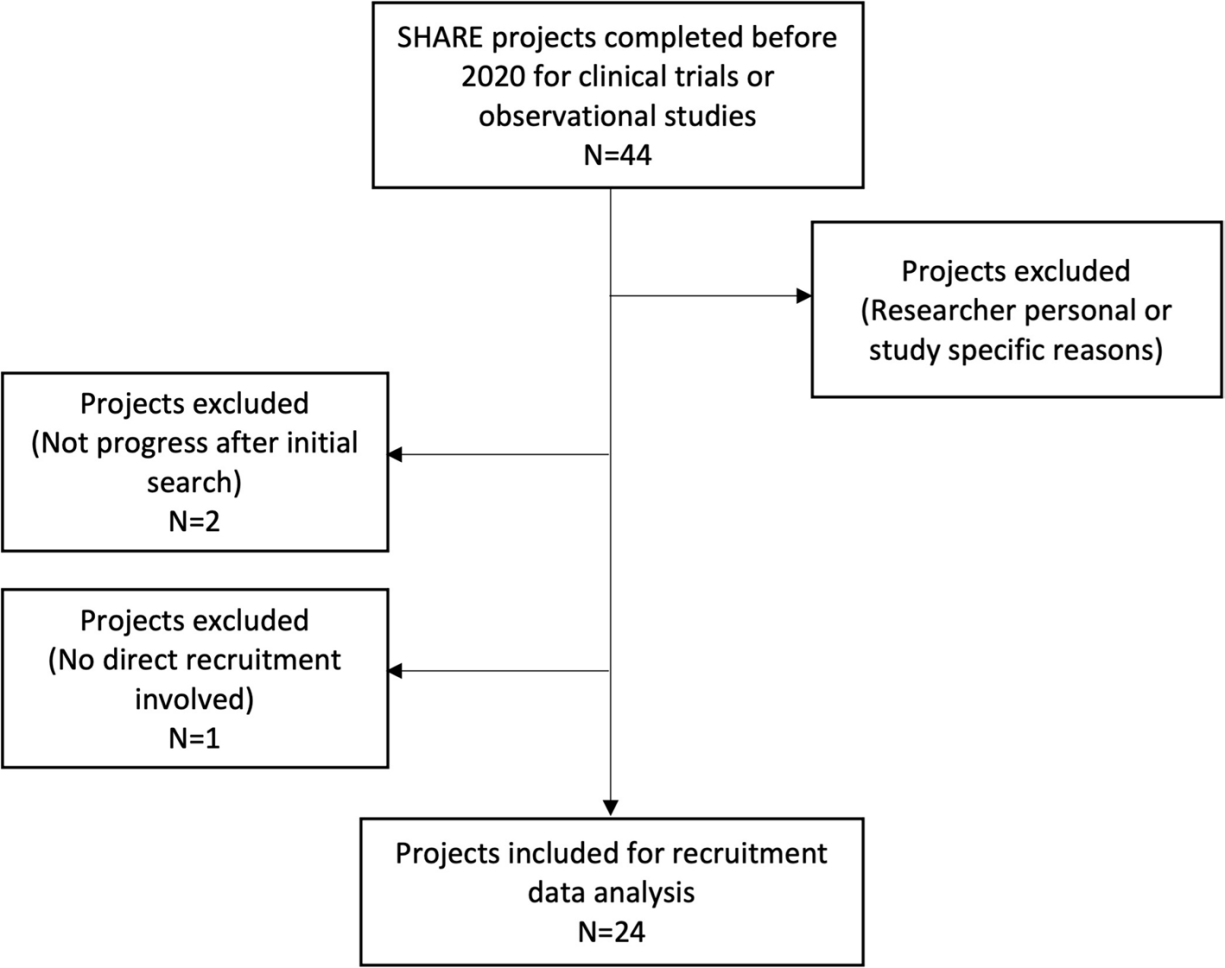
Diagram of including and excluding projects for recruitment data analysis

Figure 3 is the flow diagram of including and excluding projects for recruitment data analysis. Forty-four projects for trials or observational studies finished before 2020. They were eligible for inclusion, but 17 were excluded from recruitment data analysis due to missing records because the projects were cancelled by researchers due to personal or study specific reasons. Two projects were excluded because they did not progress after the initial search for eligible patients in the EHRs. One project was excluded because SHARE was not involved in direct recruitment of patients, so recruitment data was incomplete. Finally, 24 projects were included for recruitment data analysis.

Table 2 shows the comparison of some characteristics between included and excluded projects. Overall, excluded projects are not too different from those included in terms of time, study type, recruiting region and research domain.

**Table 2:** Comparison of characteristics between included and excluded SHARE projects

Characteristic	Included (Total *N* = 24)	Excluded (Total *N* = 20)
Year	2014: 1 2015: 1 2016: 12 2017: 6 2018: 2 2019: 2	2013: 1 2014: 2 2015: 9 2016: 4 2017: 4
Study Type	Trials: 16 Observational: 8	Trials: 18 Observational: 2
Region Involved	East: 10 Southeast: 6 West: 4 North: 1 East/West: 1 All: 2	East: 6 Southeast: 2 North: 4 East/Southeast: 1 East/North: 3 Southeast/West: 1 All: 3
Research Domain	Cardiovascular disease: 7 Diabetes: 4 Respiratory disease: 2 Alzheimer’s: 2 Mental disorder: 2 Other (gastrointestinal disease, menstruation, kidney disease, liver disease, nutrition, obesity, muscular disease): 7	Diabetes: 5 Cardiovascular disease: 4 Respiratory disease: 3 Alzheimer’s: 3 Nutrition: 2 Other (mental disorder, pain, osteoarthritis): 3

The 24 projects included recruited study subjects in various regions across Scotland. The majority (11 projects) recruited participants in the East and 2 recruited all over Scotland. Tables 3 and 4 provide details for each project and by study type respectively in terms of the region covered, the study inclusion and exclusion criteria, the number of participants requested, the number of potentially eligible individuals found through searching registrants’ EHRs, the number of people provided to researchers, the number recruited into the study and the three metrics of recruitment performance calculated. The overall trend is also presented with median and interquartile for trial and observational study separately.

**Table 3:** Study description, recruitment outcomes and three derived recruitment performance metrics for 16 clinical trials

Study Name	Region(s) Involved	Study Inclusion & Exclusion Criteria	No. Requested by researchers	No. Identified in EHRs	No. Provided by SHARE to Research Team	No. Recruited	Percentage Identified and Recruited	Percentage Provided and Recruited	Percentage Fulfilled
A	East	Inclusion: • Age: 18–75 • HF^a^ • Diabetes • On Diuretic Exclusion: • On Insulin therapy	20	68	41	11	16.2	26.8	55.0
B	Southeast	Inclusion: • Female • Age: 18–50 • Complaint of HMB^b^ • Prescription for tranexamic acid or mefenamic acid Exclusion: • Cancer • Severe Coagulation disorder • Diabetes • Hysterectomy	20	402	28	6	1.5	21.4	30.0
C	All	Inclusion: • Age:> 18 • Experienced acute coronary syndrome in the previous six months Exclusion: • Currently in hospital for any reason • Haven’t experienced acute coronary syndrome in the previous two weeks	177	NA^y^	33	16	/^z^	48.5	9.0
D	East/West	Inclusion: • Age:> 18 • CKD^c^ 3b or 4 Exclusion: • Atrial fibrillation or flutter • On warfarin • Unable to give consent • Taking Vitamin K • Known contraindication to Vitamin K • Intolerance to soya product • Pregnant or capable of child-bearing • Currently in another trial or within 30 days of completing another trial	165	237	35	7	3.0	20.0	4.2
E	Southeast	Inclusion: • Age: 40–90 • Healthy Exclusion: • Diseases of eye and adnexa • Inflammatory diseases of the central nervous system • Systemic atrophies primarily affecting the central nervous system • Extrapyramidal and movement disorders • Other degenerative diseases of the nervous system • Demyelinating diseases of the central nervous system • Vascular syndromes of brain in cerebrovascular diseases • Cerebral palsy and other paralytic syndromes • Stroke, not specified as haemorrhage or infarction	150	809	150	79	9.8	52.7	52.7
F	East	Inclusion: • LILRB5 rs12975366 genotype of either T/T or C/C • Males aged 61–69 years or Females aged 40–69 • Not on statin Exclusion: • More than 3 prescriptions in last 12 months	40	136	33	17	12.5	51.5	42.5
G	East	Inclusion: • Age:18–80 • White European • Able to complete the symptom severity score and Bristol stool chart independently • eGFR^d^ > 60 Exclusion: • Diabetes • Involvement in CTIMP^e^ within 30 days • Pregnancy or planning to conceive • Metformin history • Gastrointestinal pathology • Daily treatment with PPI^f^, anti-spasmodic, or anti-motility drugs or OCT1^g^ inhibiting drugs	40	254	8	8	3.1	100.0	20.0
H	Southeast	Exclusion: • Age:18–64 (inclusion) • have a primary DSM-5^h^ diagnosis of MDD^i^ • Not responding to at least 1 antidepressant currently taken • be able to complete study questionnaires Exclusion: • Axis I psychiatric condition • Onset of depression after 55 years of age • Organic brain disease or demented • Mental retardation • Malignancy within the 5 years • HIV • Infection of hepatitis B virus • Congestive heart failure • Demyelinating diseases • Gastrointestinal perforation	5	88	15	5	5.7	33.3	100.0
I	Southeast	Exclusion: • Age: ≥18 years old • men or post-menopausal women (women with no periods for at least 12 months or having a surgical menopause) • Hypertension Exclusion: • Ischaemic heart disease • Cardiac failure • Cerebrovascular disease • Liver impairment disease • CKD stage 3–5 • Overdose or suicidal ideation • Weight less than 55 kg • Chronic pain requiring treatment • with a known allergy to paracetamol • concomitant use of NSAIDs, oral anticoagulants or corticosteroids	20	521	36	7	1.3	19.4	35.0
J	North	Inclusion: • Males aged 18 or over • Females, postmenopausal (least 2 years without menstruation) • BMI^j^:19–35 • Healthy, free of long-term medication Exclusion: • Age: < 18 • Females, pre- or peri-menopausal (less than 2 years since last period) • Smoking • Drug abuse • Acohol > 20 units/week • Extreme diets or supplementation • Physical or mental health problems • Long-term medication	36	372	11	4	1.1	36.4	11.1
K	East	Inclusion: • Age: 18–75 • NAFLD^k^ or NASH^l^ or Hepatic fibrosis Exclusion: • Any other liver condition • Type 1 Diabetes • BMI < 25 or > 45 • Previous Bariatric surgery • History of proliferative retinopathy • History of pancreatitis • Any malignancy (except basal and squamous cell skin cancers) within the past 5 years • eGFR < 30 ml/min/1.73 m2 • On Vitamin E or pioglitazone within 90 days • On corticosteroids, methotrexate or tamoxifen or valproic acid or amiodarone, tetracycline within 28 days • HbA1c^m^ > 9% • On GLP-1^n^ RAs^o^ or SGLT-2^p^ inhibitors within 90 days	3	87	9	1	1.1	11.1	33.3
L	East	Inclusion: • Age: 18–80 • Type 2 diabetes • HF • LVSD^q^ • On Furosemide or bumetanide or torasemide Exclusion: • Age: < 18 or > 80 • Type 1 Diabetes (E10) • Chronic Liver Disease • Receiving dialysis • Malignancy (receiving active treatment) • Past or current treatment with SGLT2 inhibitor • On thiazide diuretics	26	41	13	2	4.9	15.4	7.7
M	Southeast	Inclusion: • 65 years old or older • Residents of Edinburgh or surrounding areas (including Lothians) Exclusion: • Have a history of dementia • Stroke • Other neurological disorders • Traumatic brain injury • Major psychiatric disorders	300	1000	132	69	6.9	52.3	23.0
N	East	Inclusion: • Age ≧40 and ≦80 • Age of diabetes diagnosis ≧35 • T2DM^r^/NIDDM^s^ • Metformin monotherapy • HbA1c ≦8% (64 mmol/mmol) • eGFR ≧50 ml/min-1 • ALT^t^ ≦2.5*ULN^u^ Exclusion: • Type 1 Diabetes Mellitus • Anaemia (Haemoglobin < 12.0 g/dL for women, < 13.0 g/dL for men) • Pregnancy, lactation or a female planning to conceive within the study period • Established pancreatic disease • Participating in clinical phase of another interventional trial/study or have done so within the last 30 days	20	169	22	7	4.1	31.8	35.0
O	Southeast	Inclusion: • Males and females aged 18–40 years (inclusive at selection) • Body mass index between 18.5-25 kg/m2 (lean group) or 30–55 kg/m2 (obese group) • No acute or chronic medical conditions Exclusion: • Major psychiatric illness • Recurrent depression/ cyclothymic disorder • Diabetes • Anti-diabetic drugs • Anti-depressants and mood altering drugs • Beta-blockers • Anticoagulants and antiplatelets • Glucocorticoids	18	233	32	7	3.0	21.9	38.9
P	West	Inclusion: • Male or female, between and including 40–85 years of age • A confirmed physician diagnosis of COPD^v^ • Current or ex-smoker with ≥10 pack years of smoking history. • Post bronchodilator FEV1^w^/FVC^x^ ratio < 0.7 • Post bronchodilator FEV1 ≥ 40% of the predicted value. • To have had 1 or more COPD exacerbations in the last 12 months requiring intervention with oral corticosteroids and/or antibiotics such as Penicillins, Macrolides, Tetracyclines, Quinolones, Corticosteroids • Should be prescribed and taking regularly one or more long acting bronchodilator (e.g. long acting? 2 agonist [LABA], long acting muscarinic antagonist [LAMA]) with or without an inhaled corticosteroid maintenance therapy for their COPD such as Broncodilators, Corticosteroids (for more than 6 months) Exclusion: • Active interstitial lung disease • Past history of lung cancer • Significant bronchiectasis • Cystic fibrosis • Alpha-1 antitrypsin deficiency • History of significant chronic asthma • Patients who are currently receiving anti-epileptic therapy and/or have uncontrolled epilepsy • Patients with unstable ischaemic heart disease (including, but not limited to, unstable angina • Myocardial infarction) within 6 months • Stroke within the preceding 6 months	5	496	18	2	0.4	11.1	40.0
Median (Interquartile)			20 (19–40)	235 (100–395)	28 (14–34)	7 (5–10)	3.1 (1.4–5.5)	26.8 (19.7–42.5)	33.3 (15.6–39.5)

^a^Heart Failure

^b^Heavy Menstrual Bleeding

^c^Chronic Kidney Disease

^d^estimated Glomerular Filtration Rate

^e^Clinical Trial of Investigational Medicinal Product

^f^Proton-pump inhibitor

^g^Organic cation transporter 1

^h^DSM-5: The Diagnostic and Statistical Manual of Mental Disorders, Fifth Edition

^i^Major Depressive Depression

^j^Body Mass Index

^k^Non-Alcoholic Fatty Liver Disease

^l^Nonalcoholic Steatohepatitis

^m^Hemoglobin A1c

^n^Glucagon-like peptide-1

^o^receptor agonists

^p^Sodium/Glucose Cotransporter 2

^q^Left Ventricular Systolic Dysfunction

^r^Type 2 Diabetes Mellitus

^s^Non-Insulin-Dependent Diabetes Mellitus

^t^Alanine Transaminase

^u^Upper Limits of Normal

^v^Chronic Obstructive Pulmonary Disease

^w^Expiratory volume measured during the first second of the forced breath

^x^Forced Vital Capacity

^y^missing data

^z^cannot be calculated due to missing data

**Table 4:** Study description, recruitment outcomes and three derived recruitment performance metrics for 8 observational studies

Study name	Region(s) Involved	Study Inclusion & Exclusion Criteria	No. Requested by researchers	No. Identified in EHRs	No. Provided by SHARE to Research Team	No. Recruited	Percentage Identified and Recruited	Percentage Provided and Recruited	Percentage Fulfilled
Q	West	Inclusion: • Age: 30–75 • Healthy Exclusion: • History of peptic ulcer disease • History of Barrett’s oesophagus • Previous *H. pylori* eradication • Current use of PPI^a^/H2 blockers • PPI use within last 12 months • Upper GI cancer • Pregnancy	150	1565	243	134	8.6	55.1	89.3
R	East	Inclusion: • Aged 60–85 • Fluent in English • Able to read • Reliable study partner/informant Exclusion: • Dementia or any degenerative brain disorder • Brain disease • On cholinesterase inhibitors and/or memantine • Receiving daily medications with the potential to affect cognition such as sedatives, pain medications, or anticonvulsants within 30 days • Familial autosomal dominant Alzheimer’s disease or other familial dementing diseases • Uncontrolled or untreated thyroid problems • Clinically significant vitamin B12 or folic acid deficiency • Chromosome21trisomy • History within the past 2 years or current diagnosis of significant psychiatric illness • History within the last 5 years of a serious infectious disease affecting the brain or head trauma resulting in protracted loss of consciousness • Contraindications for MRI • Major surgery, requiring general anaesthesia within 8 weeks or has not recovered • Exposure to ionizing radiation • Learning disability • Currently participating in any interventional studies • Currently participating in non-interventional study that involves exposure to radiation or neuropsychological testing • History within the last 5 years of any type of cancer • Currently on regular Vitamin B injections • Currently taking anti-seizure medications • Currently taking co-codamol or dihydrocodeine • Currently taking opioids • Currently taking benzodiazepines • Stroke • Blindness • Deafness or significant hearing impairment • Seizures	250	2365	700	91	3.8	13.0	36.4
S	All	Inclusion: • Age: ≥16 years • Diagnosis within the last 20 months of type 1diabetes or non-type 1 diabetes treated with diet non-type 1 diabetes on metformin monotherapy only • Able to give informed consent Exclusion: • Non-type 1 diabetes on any oral hypoglycaemic agent other than metformin and who have had a prescription in the last 6 months • Non-type 1 diabetes treated with an injectable therapy and who have had a prescription in the last 6 months • Gestational diabetes	6000	3828	1040	246	6.4	23.7	4.1
T	East	Inclusion: • Age: ≥70 • Heart Failure (NYHA^b^ class II to IV) • On frusemide) and ACE-i^c^ Exclusion: • Living in nursing or residential home accommodation	30	99	36	19	19.2	52.8	63.3
U	East	Inclusion: • Age: > 40 • COPD Exclusion: • Implemented during investigator screening	150	113	82	21	18.6	25.6	14.0
V	West	Inclusion: • Age: 20–39 Exclusion: • Insulin dependent diabetes • Diseases of the Nervous System 1 • Diseases of the Nervous System 2 • Congenital malformations of the nervous system • Inability to give informed consent • Mental retardation • Alzheimer’s Disease • Other degenerative diseases of the CNS^d^, not elsewhere specified • Other degenerative disorders of nervous system in diseases classified elsewhere • myotonic dystrophy or other muscular dystrophy • family history of myotonic dystrophy and never tested • Severe concurrent medical condition, e.g. cardiac failure or respiratory failure • Contraindications to MRI^e^ • History of major head trauma with loss of consciousness greater than a few minutes or with significant medical sequelae	8	66	10	7	10.6	70.0	87.5
W	East	Inclusion: • Age: 40–85 • Type 2 Diabetes • Blood pressure ≤ 140/80 mmHg • HbA1c^h^ value ≤64 mmol/mol Exclusion: • Blood pressure > 140/80 mmHg • eGFR^i^ < 60 • Atrial fibrillation • Heart failure • Peripheral vascular disease • Stroke • TIA^j^ • Ischaemic heart disease • Inability to consent	30	238	24	5	2.1	20.8	16.7
X	West	Inclusion: • Male or female ≥18 years of age • Residing in Scotland • Diagnosis of Bipolar Disorder • At least three months lithium treatment in the past or current lithium treatment	190	16	6	6	37.5	100.0	3.2
Median (Interquartile)			150 (30–190)	238 (99–1565)	82 (24–243)	21 (7–91)	9.8 (6.4–18.6)	52.7 (23.7–55.1)	36.4 (14.0–63.3)

^a^Proton-pump inhibitor

^b^the New York Heart Association Functional Classification

^c^angiotensin-converting-enzyme inhibitor

^d^central nervous system

^e^magnetic resonance imaging

^h^Hemoglobin A1c

^i^estimated Glomerular Filtration Rate

^j^Transient Ischaemic Attack

Overall, 34.2% (interquartile range 13.3–45.1%) of the number of participants requested by researchers were fulfilled through SHARE. Further analysis of the cohort provided to researchers showed that the average recruitment rate was 29.3% (interquartile 20.6–52.4%). The number of potential candidates identified by searching EHRs ranged from 94 to 509 across studies and on average there were 237 people to be further screened for eligibility for a study.

We received 12/44 responses to the online survey (response rate 27%). Two researchers chose not to reveal their study names. There were three studies of which the names reported could not be matched with those recorded at SHARE. Among the remaining seven identifiable studies, four are trials and three are observational studies.

According to the survey results, two studies used SHARE as the principal means of participant recruitment. Six reported that they adopted SHARE as a supplementary recruitment method in addition to a variety of approaches such as other patient registers, direct clinical contact, mass media, social media. The other four studies turned to SHARE after their original recruitment strategy failed. SHARE was involved within the first three months of participant recruitment for five studies and during months 3–6 for another two studies. In the other five studies, SHARE was used in the late phase of the recruitment including two engaging SHARE after one year into the study.

The participant target and recruitment outcomes of different methods reported by researchers and the study information for identifiable studies are presented in the Table 5. The recruitment performance calculated according to outcomes reported by researchers are also presented in it. The overall fraction of the number recruited through SHARE over the study target number was 31.7% (interquartile 5.8–59.6%). The recruitment rate centred at 20.2% (interquartile 8.2–31.0%) in terms of the number of candidates received. Notably, the researcher of study Ω responded “*impossible via other means*” when asked about the difficulty encountered during participant recruitment while SHARE managed to find all the participants for it.

**Table 5:** Study description, recruitment outcomes reported by researchers and two derived performance metrics

Study name	Region(s) Involved^c^	Study Inclusion & Exclusion Criteria^c^	Target No. of Participants	No. Received from SHARE	No. Recruited through SHARE	No. Recruited through Other Recruitment Means	Percentage Provided and Recruited	Percentage Fulfilled
Ω	-^f^	-^f^	20	30	20	0	66.7	100.0
V	West	Inclusion: • Age: 20–39 Exclusion: • Insulin dependent diabetes • Diseases of the Nervous System 1 • Diseases of the Nervous System 2 • Congenital malformations of the nervous system • Inability to give informed consent • Mental retardation • Alzheimer’s Disease • Other degenerative diseases of the CNS^d^, not elsewhere specified • Other degenerative disorders of nervous system in diseases classified elsewhere • myotonic dystrophy or other muscular dystrophy • family history of myotonic dystrophy and never tested • Severe concurrent medical condition, e.g. cardiac failure or respiratory failure • Contraindications to MRI^e^ • History of major head trauma with loss of consciousness greater than a few minutes or with significant medical sequelae	60	NA^d^	8	48 from clinical database of patients with myotonic dystrophy; 12 controls recruited from patients’ families	/^e^	13.3
NA^d^	-^f^	-^f^	20	NA^d^	10	10	/^e^	50.0
R	East	Inclusion: • Aged 60–85 • Fluent in English • Able to read • Reliable study partner/informant Exclusion: • Dementia or any degenerative brain disorder • Brain disease • On cholinesterase inhibitors and/or memantine • Receiving daily medications with the potential to affect cognition such as sedatives, pain medications, or anticonvulsants within 30 days • Familial autosomal dominant Alzheimer’s disease or other familial dementing diseases • Uncontrolled or untreated thyroid problems • Clinically significant vitamin B12 or folic acid deficiency • Chromosome21trisomy • History within the past 2 years or current diagnosis of significant psychiatric illness • History within the last 5 years of a serious infectious disease affecting the brain or head trauma resulting in protracted loss of consciousness • Contraindications for MRI • Major surgery, requiring general anaesthesia within 8 weeks or has not recovered • Exposure to ionizing radiation • Learning disability • Currently participating in any interventional studies • Currently participating in non-interventional study that involves exposure to radiation or neuropsychological testing • History within the last 5 years of any type of cancer • Currently on regular Vitamin B injections • Currently taking anti-seizure medications • Currently taking co-codamol or dihydrocodeine • Currently taking opioids • Currently taking benzodiazepines • Stroke • Blindness • Deafness or significant hearing impairment • Seizures	250	650	152	375	23.4	60.8
E	Southeast	Inclusion: • Age: 40–90 • Healthy Exclusion: • Diseases of eye and adnexa • Inflammatory diseases of the central nervous system • Systemic atrophies primarily affecting the central nervous system • Extrapyramidal and movement disorders • Other degenerative diseases of the nervous system • Demyelinating diseases of the central nervous system • Vascular syndromes of brain in cerebrovascular diseases • Cerebral palsy and other paralytic syndromes Stroke, not specified as haemorrhage or infarction	150	156	84	3 friends of people who had agreed to help during previous study	53.8	56.0
Y	West and Southeast	Inclusion: • Diagnosis of F20.0, F20.1, F20.2, F20.3, F20.5, F20.9 • 25–55 years of age • Body Mass Index (BMI) of 18 to 30 kg/m2 • A total body weight 50–100 kg • Stable Antipsychotic medication for 1 month prior to visit 1 Exclusion: • An existing neurological disorder • Metal implants in body parts • Pregnancy • Psychiatric hospitalization over 6 months before visit 1 • Change of medication ≤1 month prior to visit 1 • Patients who are currently taking clozapine • Current substance abuse, including cannabis	30	30–50	0	2	0	0
B	Southeast	Inclusion: • Female • Age: 18–50 • Complaint of HMB^b^ • Prescription for tranexamic acid or mefenamic acid Exclusion: • Cancer • Severe Coagulation disorder • Diabetes • Hysterectomy	108	28	6	85 through NHS Menstrual Problem; 38 through GP letter by SPCRN^a^ or leaflet given by GP; 20 through poster in clinical area, NHS/University staff website; 27 from Facebook adverts	21.4	5.6
NA^d^	/^e^	/^e^	350	NA^d^	NA^d^	NA^d^	/^e^	/^e^
I	Southeast	Exclusion: • Age: ≥18 years old • men or post-menopausal women (women with no periods for at least 12 months or having a surgical menopause) • Hypertension Exclusion: • Ischaemic heart disease • Cardiac failure • Cerebrovascular disease • Liver impairment disease • CKD stage 3–5 • Overdose or suicidal ideation • Weight less than 55 kg • Chronic pain requiring treatment • with a known allergy to paracetamol • concomitant use of NSAIDs, oral anticoagulants or corticosteroids	110	37	7	100 through SPCRN; 97 through ABPM^b^ clinics	18.9	6.4
Σ	-^f^	-^f^	12	92	10	13	10.9	83.0
Φ	-^f^	-^f^	100	0	0	103	0	0
M	Southeast	Inclusion: • 65 years old or older • Residents of Edinburgh or surrounding areas (including Lothians) Exclusion: • Have a history of dementia • Stroke • Other neurological disorders • Traumatic brain injury • Major psychiatric disorders	300	NA^d^	NA^d^	NA^d^	/^e^	/^e^

^a^Scottish Primary Care Research Network

^b^Ambulatory blood pressure monitoring

^c^data come from study records held by SHARE, not from the survey responses

^d^missing, not provided by respondents

^e^cannot be calculated due to missing data

^f^cannot be retrieved because the study name reported by researchers is unidentifiable

Seven researchers (58.3%) agreed that the participants transferred to them by SHARE were either more eligible or about the same as those found through other methods. Eight respondents (66.7%) considered SHARE a quicker way to recruit participants compared with other means of recruitment. Whereas 58% of those surveyed gave less favourable responses citing the cost of recruiting through SHARE.

When asked for suggestions for improvement, the most frequently raised issue (mentioned by 3 respondents) can be summarized as the need to improve the eligibility of the candidates transferred. Researchers hoped to receive as few ineligible candidates as possible so that they would be able to save some resources on filtering out ineligible participants. Other than that, one of the researchers was very discontent with the cost charged by SHARE. One researcher requested more rapid responses from the SHARE team. Another researcher mentioned improving the ability to identify mild cognitive impairment. One respondent encouraged SHARE to continue enlarging the registrant base.

The raw data obtained from the survey are attached as Additional file 2.

## Discussion

Based on the recruitment data at SHARE, the register can provide over one third of the number of participants requested by study teams on average. Generally, one eligible participant is recruited for every 3 to 4 candidates provided to researchers by SHARE. The recruitment performance derived from the survey is a little poorer compared to that from the SHARE data (31.7 and 20.2% regarding target fulfilment and effective recruitment of candidates provided respectively) and with more variation. The percentage fulfilment reported by researchers may seem lower since it was calculated against the total target number of a study rather than the number SHARE was requested for. It reflected how much SHARE contributed to the recruitment outcome of a study, but it might be an underestimate because researchers might have already recruited most of the participants needed through other methods then they wouldn’t request that many from SHARE. Overall, most researchers consider SHARE a quicker way to recruit participants and the candidates provided by SHARE same likely to be eligible for recruitment as other methods if not more.

SHARE has shown relatively good performance compared with other recruitment methods for most studies reported in the survey. Researchers need to screen 5 candidates from SHARE for one successful recruit on average and 75% screen fewer than 12. This recruitment performance is still encouraging compared to those reported by other studies. For example, a cardiovascular disease trial reported to screen 5 candidates per participant recruited with the method of offering £100 upon successful recruitment at first place [26]. The number of candidates screened in some other studies ranged between 8 and 26 adopting one or more recruitment strategies such as small financial incentive, church involvement, telephone reminder, text message [27–30].

There are several studies for which SHARE seems to have poorer performance than other recruitment methods, i.e., study V, study B, study I, study Φ. But these studies were reported to involve SHARE in their recruitment at a late stage, and all but study V claimed that other recruitment strategies were failing, which to some extent indicates the extreme difficulty of recruitment for these studies. Considering SHARE was approached later in the recruitment phase of these studies, it is understandable that SHARE contributed fewer participants than other methods. On the contrary, according to the data held by SHARE, the recruitment performance regarding the number of participants requested for study V was about 87%, which is quite good. However, studies which had highly restrictive inclusion and exclusion criteria posed a challenge when those criteria cannot be mapped to structured EHR data. SHARE mainly use hospital admissions data to identify disease status but some conditions are either not coded in the EHRs or incompletely coded such as menstrual bleeding required by study B and mild cognitive impairment required by study Φ. The relatively poor performance in study I is probably due to the reason that most hypertensive patients captured in secondary care data suffer from comorbidity which made them ineligible according to the exclusion criteria of the study and mild hypertensive patients are mostly seen by their general practitioners (GPs). Therefore, SHARE is unable to identify these patients from secondary care data and find as many eligible patients for researchers as they wanted for their study.

To solve these problems, it is important that SHARE access EHRs generated from as many patients’ health care encounters as possible including diagnosis during primary care, in-hospital medication records, discharge notes, etc. This modification would also contribute a solution to researchers’ request for more quality control on the eligibility of the candidates provided. Now, whether the cohort built from EHRs meet researchers’ criteria depends hugely on what kind of condition is under consideration, whether it is coded in hospital admissions data and how accurately it is recorded. Data currently in use are mainly coded according to International Classification of Diseases (ICD). ICD codes are widely criticized for lack of granularity or aetiological information and poor accuracy for identification of certain diseases [31]. There are a few studies which have proved that the accuracy of pre-screening can be significantly increased and workload can be reduced by using both clinical narrative data and coded data to search for potentially eligible candidates [31–34]. Incorporating more data sources is likely to overcome many of those issues and help SHARE improve. According to the SHARE team, primary care data is now in the process of being added to the data inventory.

The limitations of this study include the fact that the major source of the recruitment data was the SHARE management team and not all studies were analysed for recruitment performance so there might exist selection bias of providing data for studies with relatively good results. We additionally conducted a survey involving the research teams of all the eligible projects. Recruitment data collected from the survey complemented the evaluation of recruitment performance based on SHARE records. Another limitation of the study is the seemingly low survey response rate, though a response rate of 20–30% is considered acceptable for an online survey according to SurveyMonkey [35]. The survey was meant to let researchers vent their real thoughts without worry of being identified and their future studies being affected because of them giving negative feedback. Although the response rate was not too high, it was designed and conducted in a stringent way. We believe that we have successfully gathered miscellaneous researchers’ views on the effect of the service on their studies’ recruitment performance and have drawn valuable information from them. Other survey methods may have resulted in even lower response rates.

## Conclusions

In conclusion, SHARE may be a valuable resource for recruiting participants for some clinical studies. Currently it has blind spots in identifying some symptoms and minor ailments due to the intrinsic lack of access to the data reflecting those healthcare encounters. With the continuing growing number of registrants and the potential of eased restriction of data access, SHARE can become an even more powerful tool for efficient and effective participant recruitment to health research.

## Supplementary Information

**Additional file 1.** Protocol_question_pis.docx. This file contains the protocol of the study, the survey questionnaire and the participant information sheet.

**Additional file 2.** SurveyRaw.xlsx. Raw survey data.

## Data Availability

The recruitment data analysed in the current study are not publicly available because it also contained study and researcher information. But Ms. Shobna Vasishta can be contacted through s.vasishta@dundee.ac.uk for enquiry about access to the recruitment data. The survey data analysed in the current study are attached as Additional file 2 and all the study names in it were masked.

## Abbreviations

SHARE: The Scottish Health Research Register
EHRs: Electronic Health Records
CTUs: Clinical trials units
HIC: The Health Informatics Centre
GPs: General practitioners
ICD: International Classification of Diseases
