# Supplementary material for: Early experience with an opt-in research register - Scottish Health Research Register (SHARE): a multi-method evaluation of participant recruitment performance
Source: BMC Med Res Methodol. 2021 Dec 20;21:286. doi: 10.1186/s12874-021-01479-4 (PMC8686271; doi:10.1186/s12874-021-01479-4)
Supplement: Supplementary file 1 — Additional file 1. Protocol_question_pis.docx. This file contains the protocol of the study, the survey questionnaire and the participant information sheet. [file 12874_2021_1479_MOESM1_ESM.docx]

**Protocol of a study to survey researchers who have recruited participants through SHARE**

**Background**

Recruiting participants for clinical studies is time-consuming and resource-intensive especially for clinical trials. Poor recruitment can upset and demoralize researchers, prolong the time of recruitment and place larger burden on resources.[1] However, a study in 2013 revealed that trials in the UK were struggling to achieve the target sample size with a rate of target achievement of 55% and a time-extension for funding of 45%.[2] Tudur Smith et al. conducted an on-line Delphi survey of 48 clinical trials units (CTUs) and identified ‘*Research into methods to boost recruitment in trials*’ as the top priority in trial methodological research.[3] Although the difficulty in participant recruitment and its crucial ramification has been well-recognized and frequently-stressed, strategies to deal with this problem tend to be rare and contain insufficient evidence.

The Scottish Health Research Register (SHARE)[4] seeks consent from people across Scotland for their Electronic Health Records (EHRs) to be utilized for searching clinical study participant, which has more than 250,000 registrants now. The datasets currently available and normally used for query are from hospital admissions, community prescriptions, laboratory, cancer registry. SHARE has, since inception, been used as the sole method of recruitment for some studies and it has assisted recruitment of several studies that failed to recruit participants by using traditional recruitment methods.[5]

Analysis of the recruitment performance of 25 SHARE projects shows that the median recruitment rate was 42.5% (interquartile 16.7% - 66.7%) of the number of participants requested by each study. 36.4% (interquartile 20.8%-55.1%) of the candidates passed on to the researchers were recruited. SHARE recruited more than half of the participants requested for 12 studies which also include three studies having found all those needed. However, the recruitment results were less satisfactory among the other 13 studies. Overall, SHARE did best with studies carrying out surveys (median recruitment rate 74.6%, interquartile 61.7%-95.6%). Nevertheless, the median recruitment rates in terms of the numbers requested dropped to 44.5% (interquartile 16.0% - 69.4%) and 30.0% (interquartile 10.1% - 38.8%) for observational studies and trials respectively. By contrast to the high enrolment in people transferred to researchers of surveys, the figures were far less optimistic for observational studies (median 39.1%, interquartile 20.8% - 53.4%) and trials (median 26.8%, interquartile 19.7% - 42.4%).

However considering the high risk of failure and the constraint of time and resources clinical trial face during participant recruitment, SHARE is still likely to be an extremely valuable approach. SHARE’s approach is also being adopted by research support groups elsewhere.[6, 7] In order to evaluate the recruitment performance of SHARE within the context of each particular study and continually improve SHARE, a survey of the study researchers who have used SHARE for recruitment is proposed as following.

**Aim**

To understand the role that SHARE has played in recruitment to a range of trials and observational studies between initiation and 2019 and its relative performance and how researchers rate participant recruitment through SHARE in terms of effectiveness and efficiency.

**Objectives**

- To describe the role SHARE has played in the recruitment phase
- To compare recruitment performance between different approaches
- To list researchers’ feedback and suggestions for improvements

**Method**

The survey will be implemented using an open source online tool - Qualtrics. The link will be sent to researchers by email (see supplement file for the list of researchers). The survey is meant to be brief so it won’t take up researchers’ too much time. Two weeks after the first email, a reminder will be sent by email. The online survey is designed as follows:

Opening Statement

You are being invited to participate in a research study titled ‘An online survey of researchers who have recruited participants through the Scottish Health Research Register (SHARE)’. This study is being done by Wen Shi from the School of Medicine at the University of St Andrews.

The researcher tries to understand the role that SHARE has played in participant recruitment to clinical research and its relative performance and hopes to gain knowledge from your experience of recruiting participants to your research project and your recruitment outcomes, thus helping improve the effectiveness and efficiency of recruiting through SHARE. You will be asked to complete a questionnaire which contains 13 questions. Some of the questions might need you to look up your study management file for recruitment outcomes. We’d really appreciate it if you could provide us with as accurate data for those questions as possible. Further details, including information about data protection, are available in the participant information sheet attached to the email sent to you.

If you are interested in taking part, please read the participant information sheet and keep a copy before starting the survey. If you have any questions, please email me at [ws51@st-andrews.ac.uk](mailto:ws51@st-andrews.ac.uk).

Your participation is entirely voluntary, and you can withdraw at any time.

By clicking the ‘Submit’ button in the last page, you will be consenting to participate in this study.

Questions

1. Study name (identifiable)
2. What was the target number of participants for the study using all recruitment methods?
3. How was SHARE used for recruiting participants to the study?
4. The main means of participant recruitment
5. A supplement to other means of participant recruitment
6. As an additional recruitment method when other methods were proving insufficient.
7. What recruitment methods were used other than SHARE?
   1. By contacting patients on a register of patients
   2. Through clinical contact with potential study subjects
   3. Media e.g. newspapers
   4. Others please specify
8. Were any difficulties encountered using other methods of recruitment?
9. What was the number of potential participants sent to you from SHARE? (maybe identifiable)
10. What was the number of participants recruited through SHARE? (maybe identifiable)
11. What was the number of participants recruited through other methods (please list the number recruited according to different recruitment methods)?
12. At what stage of the recruitment phase did you involve SHARE?
13. 1st 3 months
14. 3-6 months
15. 6 months -end of study
16. Others please specify
17. How likely to be eligible for your study were the potential participants about whom SHARE notified you?
    1. More likely to be eligible than other methods
    2. Less likely to be eligible than other methods
    3. About the same as other methods
18. Was it quicker to recruit participants through SHARE compared with other methods?
    1. Yes
    2. No
    3. About the same as other methods
19. Was it more cost-effective to recruit participants through SHARE compared with other methods?
20. Yes
21. No
22. About the same as other methods
23. Do you have any suggestions for improving the effectiveness and efficiency of recruiting through SHARE?

Closing Statement

By clicking the ‘Submit’ button below, you are consenting to participate in this study, as it is described in the participant information sheet, which was sent to you by email. If you did not yet download and keep a copy of this document for your records, we recommend you do that now.

**References**

1. Prescott R, Counsell C, Gillespie W, Grant A, Russell I. Health Technology Assessment. 1999;3(20).

2. Sully BGO, Julious SA, Nicholl J. A reinvestigation of recruitment to randomised, controlled, multicenter trials: a review of trials funded by two UK funding agencies. Trials. 2013;14:166. DOI: 10.1186/1745-6215-14-166.

3. Tudur Smith C, Hickey H, Clarke M, Blazeby J, Williamson P. The trials methodological research agenda: results from a priority setting exercise. Trials. 2014;15:32. DOI:10.1186/1745-6215-15-32.

4. The Scottish Health Research Register[Internet]. Available from: [www.registerforshare.org.uk](file:///C:\Users\ws51\Documents\share%20survey\www.registerforshare.org.uk). Accessed Oct 2019.

5. McKinstry B, Sullivan FM, Vasishta S, Armstrong R, Hanley J, Haughney J, et al. Cohort profile: the Scottish Research register SHARE. A register of people interested in research participation linked to NHS data sets. BMJ Open [Internet]. 2017 Feb; 7(2). Available from: <http://bmjopen.bmj.com/content/7/2/e013351.long>. Accessed July 2019. DOI:10.1136/bmjopen-2016-013351.

6. The “All of Us” Research Program. N Engl J Med. 2019;381(7):668-76. DOI:10.1056/NEJMsr1809937.

7. Imperial College Health Partners. Discover-NOW [Internet]. Available from: <https://imperialcollegehealthpartners.com/what-we-do/discover-now/>. Accessed Oct 2019.


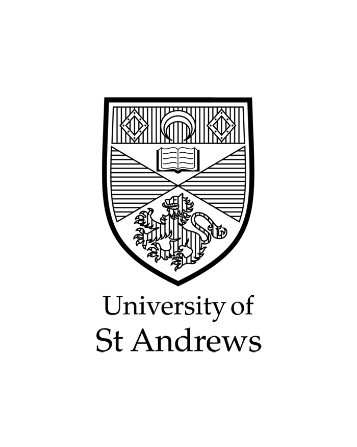
Participant Information

**An online survey of researchers who have recruited participants through the Scottish Health Research Register (SHARE)**

**Wen Shi**

What is the study about?

We invite you to participate in a research project trying to understand the role that SHARE has played in participant recruitment to clinical research and its relative performance. We plan to survey 44 studies’ chief investigators and study coordinators through online survey tool - Qualtrics. We hope to gain knowledge from your experience of recruiting participants to your research project and your recruitment outcomes and help improve the effectiveness and efficiency of recruiting through SHARE.

Why have I been invited to take part?

According to SHARE, you used it as one participant recruitment means for your research project. That’s why we sincerely invite you to take part in this survey study and share with us your experience of participant recruitment.

Do I have to take part?

This information sheet has been written to help you decide if you would like to take part. It is up to you and you alone whether you wish to take part. If you do decide to take part you will be free to withdraw at any time during online data collection without providing a reason, and with no negative consequences. Even after your submission of the survey online, you will be able to withdraw your data within 30 days, but only if you answered the identifiable question in the questionnaire, because we won’t be able to identify your data otherwise.

What would I be required to do?

You will be asked to complete a questionnaire which contains 13 questions regarding participant recruitment details of your study and your opinions on using SHARE as a recruitment tool. You may not have accurate data for some questions such as recruitment results, but please be as precise as possible. We would really appreciate it if you could provide us with as accurate data for those questions as possible.

Informed consent

By completing the survey and submitting it online, you give your informed consent to participation and being in full knowledge of the information in the participant information sheet. You can ask any question in relation to the research before or during the survey.

Who is funding the research?

My research is not funded. It will contribute to my PhD project of studying how to improve participant recruitment into clinical research through using electronic health records.

What information about me or recordings of me (‘my data’) will you be collecting?

All the questions are about participant recruitment of your study and your opinions on using SHARE as a means of recruiting participants. You will be able to answer them through a reliable online survey tool-Qualtrics. It helps keeping data anonymous. However, we ask the study name (Q1) in the questionnaire so that we can link the recruitment results with a particular study and further analyse the relationship between the study characteristics and the recruitment results. By doing so, we hope it can better answer our research question. But if you would like to maintain anonymous, you can omit this question. There are another two optional questions regarding recruitment results of using SHARE (Q6, Q7) which we think you might not want to answer if you would like to avoid indirect identification.

How will my data be securely stored, who will have access to it?

Researchers will be given the link to the survey, and data will be collected and stored anonymously. Note that answers to some questions may have the potential to identify a particular study but no effort will be made to do so. All data will be kept confidential. If you have any concerns about the anonymity of your answers, please contact the researcher. The data on Qualtrics will only be accessed and downloaded through the university network. After the completion of data collection, the data will be exported and stored on a password protected hard drive with limited access only by me and my supervisor. They will also be backed up on Microsoft OneDrive which can only be accessed through my university account. Data stored under my Qualtrics account will be deleted immediately following downloading.

How will my data be used, and in what form will it be shared further?

Your research data will be analysed as part of my research study. They will then be published in my thesis and potentially some selected research publication. They will also be shared with SHARE team for them to reflect on how to make improvement. **If you choose to omit all the identifiable and potentially identifiable questions**, your data shared (published and/or placed in a database accessible by others) will be in an **ANONYMOUS** form, because we and no one could use any reasonably available means to identify you from the data. **If you are willing to respond to these questions**, we will greatly appreciate it because we will be able to further analyse study characteristics and recruitment results. Data can be published and shared in an **IDENTIFIABLE form given your permission**. Otherwise they will be **ANONYMISED** according to your choice before sharing and publication.

When will my data be destroyed?

It is expected that the project to which this research relates will be finalised by Nov, 2020.

Data stored under my Qualtrics account will be deleted immediately following downloading. The data held by us will be destroyed one year after thesis submission.

Will my participation be confidential?

Yes, we will do our best to protect your confidentiality. Your data will be completely anonymous if you choose to omit the identifiable questions. However, we will be most appreciative if you would answer all the questions in the questionnaire. The answers to the identifiable questions won’t be shared or published in an identifiable form without your permission.

Use of your personal data and data protection rights

The University of St Andrews (the ‘Data Controller’) is bound by the UK 2018 Data Protection Act and the General Data Protection Regulation (GDPR), which require a lawful basis for all processing of personal data (in this case it is the ‘performance of a task carried out in the public interest’ – namely, for research purposes) and an additional lawful basis for processing personal data containing special characteristics (in this case it is ‘public interest research’). You have a range of rights under data protection legislation. For more information on data protection legislation and your rights visit <https://www.st-andrews.ac.uk/terms/data-protection/rights/>. For any queries, email [dataprot@st-andrews.ac.uk](mailto:dataprot@st-andrews.ac.uk).

You will be able to withdraw your data within 30 days after your submission of the survey online, but only if you answered the identifiable question in the questionnaire, because we won’t be able to identify your data otherwise.

Ethical Approvals

This research proposal has been scrutinised and subsequently granted ethical approval by the University of St Andrews Teaching and Research Ethics Committee.

What should I do if I have concerns about this study?

In the first instance, you are encouraged to raise your concerns with the researcher. However, if you do not feel comfortable doing so, then you should contact my Supervisor or School Ethics Contact (contact details below). A full outline of the procedures governed by the University Teaching and Research Ethics Committee is available at <https://www.st-andrews.ac.uk/research/integrity-ethics/humans/ethical-guidance/complaints/>.

Contact details

| **Researcher(s)** | Wen Shi | **Supervisor(s) / School Ethics contact** | Frank Sullivan |
| --- | --- | --- | --- |
|  | [ws51@st-andrews.ac.uk](mailto:ws51@st-andrews.ac.uk) |  | [fms20@st-andrews.ac.uk](mailto:fms20@st-andrews.ac.uk) |
|  |  |  | 01334 463512 |
